# PIC&RUN: An integrated assay for the detection and retrieval of single viable circulating tumor cells

**DOI:** 10.1038/s41598-019-53899-4

**Published:** 2019-11-25

**Authors:** Mohamed Kamal, Shahin Saremi, Remi Klotz, Oihana Iriondo, Yonatan Amzaleg, Yvonne Chairez, Varsha Tulpule, Julie E. Lang, Irene Kang, Min Yu

**Affiliations:** 1 Department of Stem Cell Biology and Regenerative Medicine, Keck School of Medicine of the University of Southern California, Los Angeles, CA 90033 USA; 2 USC Norris Comprehensive Cancer Center, Keck School of Medicine of the University of Southern California, Los Angeles, California 90033 USA; 3 Department of Zoology, Faculty of Science, University of Benha, Benha, Egypt; 4 MS Biotechnology program, California State University Channel Islands, Camarillo, CA 93012 USA; 5 Department of Medicine, Keck School of Medicine of the University of Southern California, Los Angeles, California 90033 USA; 6 Department of Surgery, Keck School of Medicine of the University of Southern California, Los Angeles, California 90033 USA

**Keywords:** Breast cancer, Metastasis

## Abstract

Circulating tumor cells (CTCs) shed from solid tumors can serve as a minimally invasive liquid biopsy for monitoring disease progression. Because CTCs are rare and heterogeneous, their biological properties need to be investigated at the single cell level, which requires efficient ways to isolate and analyze live single CTCs. Current methods for CTC isolation and identification are either performed on fixed and stained cells or need multiple procedures to isolate pure live CTCs. Here, we used the AccuCyte-RareCyte system to develop a Protocol for Integrated Capture and Retrieval of Ultra-pure single live CTCs using Negative and positive selection (PIC&RUN). The positive selection module of PIC&RUN identifies CTCs based on detection of cancer surface markers and exclusion of immune markers. Combined with a two-step cell picking protocol to retrieve ultrapure single CTCs, the positive selection module is compatible for downstream single cell transcriptomic analysis. The negative selection module of PIC&RUN identifies CTCs based on a live cell dye and the absence of immune markers, allowing retrieval of viable CTCs that are suitable for *ex vivo* culture. This new assay combines the CTC capture and retrieval in one integrated platform, providing a valuable tool for downstream live CTC analyses.

## Introduction

Cancer is one of the leading causes of death worldwide and the majority of these mortalities are caused by distant metastases^1^. Hematogeneous metastasis is a multi-step process that requires the entry of cancer cells from the primary tumor into the circulation, making circulation the interface between the primary tumor and the secondary site of metastasis^2^. Circulating tumor cells (CTCs) present in the bloodstream contain the seeds of metastasis and are considered a valuable minimally invasive liquid biopsy for solid tumors^3–6^. Molecular and functional characterization of CTCs will lead to a better understanding of metastasis and facilitate the identification of treatments that most effectively target the evolving solid tumors.

Compared to normal blood cells, CTCs are rare—usually a single digit number of CTCs is present in one milliliter (ml) of blood that also contains 10 million white blood cells (WBCs) and one billion red blood cells (RBCs). Many technologies have been developed for the identification and enumeration of CTCs^7–15^ (reviewed in^16^). These technologies enrich CTCs based on their unique physical, morphological and biological properties. Identified CTCs are typically validated by assessing mRNA or protein expression of cancer specific markers on fixed cells, which does not allow expansion of CTCs for further downstream studies.

Although technically challenging, a number of promising technologies have been developed to isolate viable CTCs^10,17–20^. The recently reported microfluidic technology, CTC-iChip, can efficiently deplete normal blood cells, enables the sorting of viable CTCs in solution^21^, and allowed the *ex vivo* culture of CTCs from 6 breast cancer patients^22^. Sufficient amount of material from these cultured CTCs enabled RNA sequencing, mutation detection, tumorigenicity analysis, as well as drug sensitivity tests. This study shows that culturing CTCs from patients provides an opportunity to study tumor biology and drug susceptibility that is unique to individual patient^22^.

In addition, since CTCs can contain tumor cells shed from multiple active tumor lesions, they have the potential to help address the complexity of intra-patient tumor heterogeneity. It has been shown that CTCs present a high degree of heterogeneity in their mutational and transcriptional profiles, as well as physical status of single cells or clusters^23–33^. Understanding CTC heterogeneity will have a profound impact on our understanding of the mechanisms of metastasis and treatment resistance. However, to unravel such heterogeneity, we need to have the tools to efficiently isolate viable CTCs individually in order to molecularly and functionally characterize them at a single cell level.

Currently, to isolate single live CTCs, additional purification steps, such as the DEPArray^34,35^, Fluidigm C1^36–39^, ALS cell-Selector^40^ or single-cell micro-manipulation, are typically used. These procedures often require additional live staining for cancer cell surface markers (CSMs), such as EpCAM, HER2 and EGFR^23^, which enable pure CTCs to be retrieved for single cell RNA-sequencing analysis^34,36,37^. However, these additional steps may lead to CTC loss and can be time-consuming. In addition, although viable CTCs isolated using these positive live markers are suitable for molecular analyses, they may not be suitable for *ex vivo* culture as the effects of antibodies on cell survival and proliferation are unclear. Therefore, there is a necessity to develop an integrated and unbiased system that allows for the isolation of single viable CTCs for single cell molecular analysis and *ex vivo* expansion.

Recently, the AccuCyte-RareCyte system was described for the identification and isolation of single CTCs. In this method, nucleated cells from a blood sample were collected using the AccuCyte sample preparation system, spread onto slides and stained with cancer cell and WBC specific antibodies. The slides were scanned by a high-speed fluorescence scanner, the CyteFinder. Finally, CTCs were retrieved using the CytePicker module, which uses a needle with a ceramic tip^41^. Although it is a very promising approach for the detection and retrieval of single fixed CTCs, it is not suitable for downstream analyses that require live cells.

**Figure 1:**
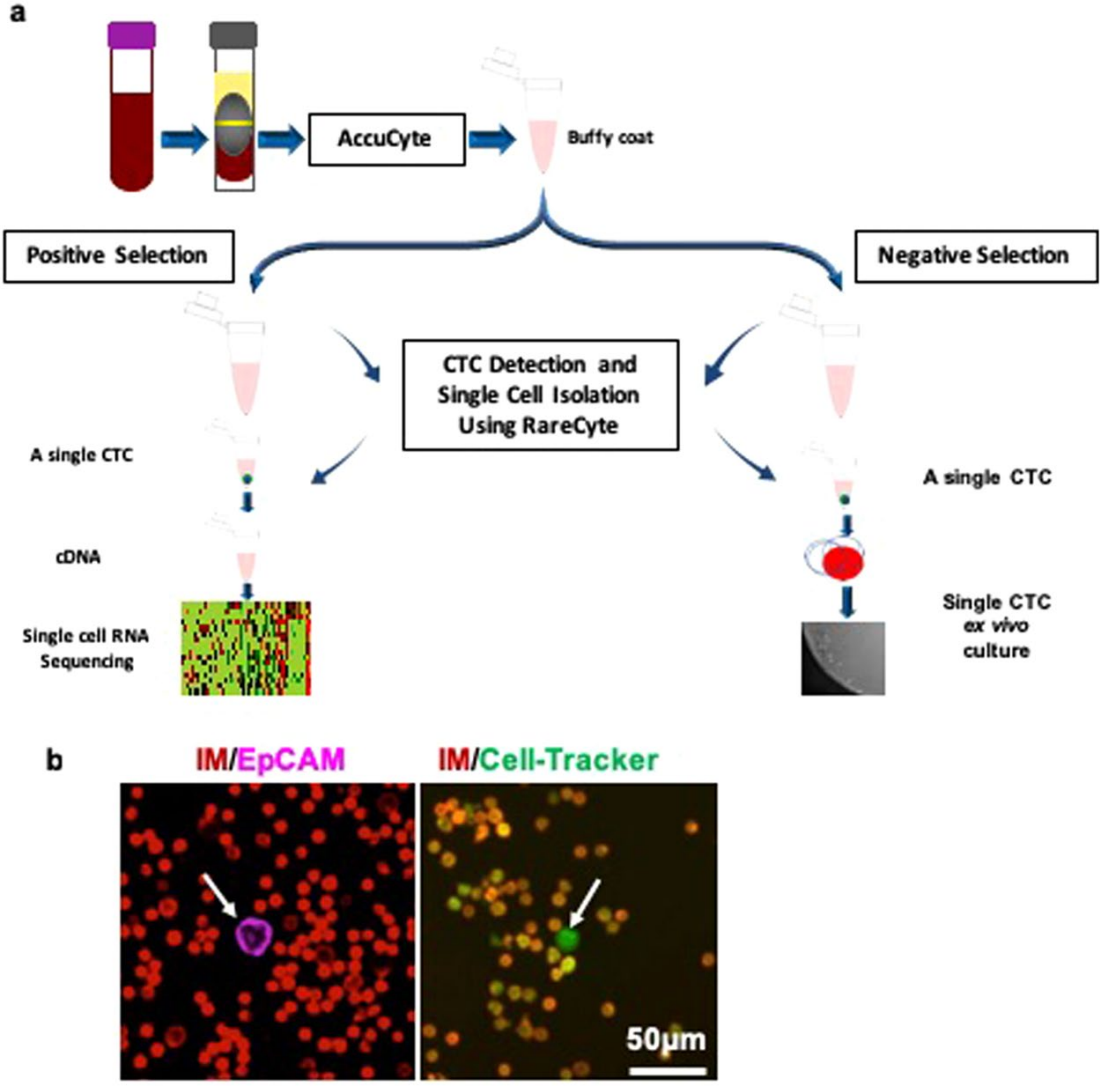
Development of PIC&RUN system. (**a**) An illustration of the PIC&RUN assay. A tube of 7.5 ml blood was processed via AccuCyte and the buffy coat was collected. Based on the planned downstream analyses, either positive or negative selection was used. Positive selection is compatible with single cell RNA sequencing analysis, whereas negative selection is compatible with *ex* *vivo* culture of single CTCs. (**b**) CTC detection based on positive or negative selection methods. Left image is a field of view of a buffy coat processed by positive selection approach with IM antibodies (red) and EpCAM antibodies (magenta). A CTC is defined as a cell with IM^−^/EpCAM^+^ (arrow). Right image is a field of view of a buffy coat processed by negative selection approach with IM antibodies (red) and Cell-Tracker green (green). A CTC is defined as a cell with IM^−^/Cell-Tracker green^+^ (arrow).

In this study, we developed a Protocol for Integrated Capture and Retrieval of Ultra-pure single live CTCs using Negative and positive selection (PIC&RUN) based on the AccuCyte-RareCyte system. If transcriptomic analyses are required, samples are processed for the positive selection module based on CSMs, whereas, if *ex vivo* culture and functional analyses are required, samples are processed using negative selection module based on exclusion of the normal blood cell markers (Fig. 1a).

## Results and Discussion

### High capture efficiency of live CTCs by accucyte

First, we used our previously established patient-derived CTC lines^22^ to test the efficiency of AccuCyte for capturing viable CTCs. CTCs (range between 165–1209) stained with the live stain DiO were spiked into 7.5 ml of blood from healthy volunteers and processed using AccuCyte. DiO positive cells from the buffy coats were counted under a fluorescence phase contrast microscope. Capture efficiency of live CTCs reached an average of 91.6% (Table 1), consistent with the previously reported capture efficiency (more than 90%) for fixed CTCs in AccuCyte-RareCyte system^41,42^.

**Table 1:** Capture efficiency of AccuCyte for live CTCs.

	ExpNo.	No. Spiked CTCs	No. Detected CTCs	CTC capture (%)
	1	165	118	71.5
	2	337	379	112.5
	3	1209	1099	90.9
Average		570	532	91.6
SD				17

### Optimization of markers for positive and negative detection of CTCs

We developed two strategies for detecting live CTCs from AccuCyte buffy coats. For both strategies, buffy coats were stained live with a cocktail of antibodies against immune markers (IM) plus either a combination of antibodies against CSMs for the positive selection approach or a live cell dye (LCD) for the negative selection approach.

We tested the sensitivity and specificity of three IM antibodies (CD14, CD16, and CD45), either individually or combined, using buffy coats from healthy volunteers’ blood. A cocktail of the three IM antibodies showed positive staining in 100% of immune cells but negative in the BRx68 CTC line (Supplementary Fig. 1).

A previously described antibody cocktail for positive selection includes antibodies against EpCAM, HER2 and EGFR^23,43^. Notably, all our CTC lines tested (BRx68, BRx07, and BRx50) showed 100% positivity for EpCAM staining. Therefore, we used EpCAM expression and absence of IM signals to detect CTCs from our CTC lines during optimization of this method (Fig. 1b). For negative selection, three LCDs—DiO, Cell-Tracker green and ViaFluor—were tested for optimum concentration, visualization and cell viability. There was no significant difference in cell proliferation between control unstained cells and any of the dyes (Supplementary Fig. 2). We decided to use Cell-Tracker green based on the optimal fluorescence intensity and degree of variability (Supplementary Fig. 3). Therefore, in the negative selection, CTCs were identified based on the lack of IM expression and the presence of Cell-Tracker green staining (Fig. 1b).

### Specificity and sensitivity of positive and negative detection protocols

To test the detection rates of both positive and negative selection approaches, spike-in experiments were performed using our EpCAM^+^ CTC lines. In order to confirm the identity of CTCs, we spiked in cells that had been pre-stained with Cell-Tracker green for the positive selection method and EpCAM for the negative selection method. Blood was then processed according to the positive selection or negative selection protocols and stained buffy coats were plated on polyhema-coated cyteslides and scanned semi-automatically using RareCyte fluorescence scanner.

**Figure 2:**
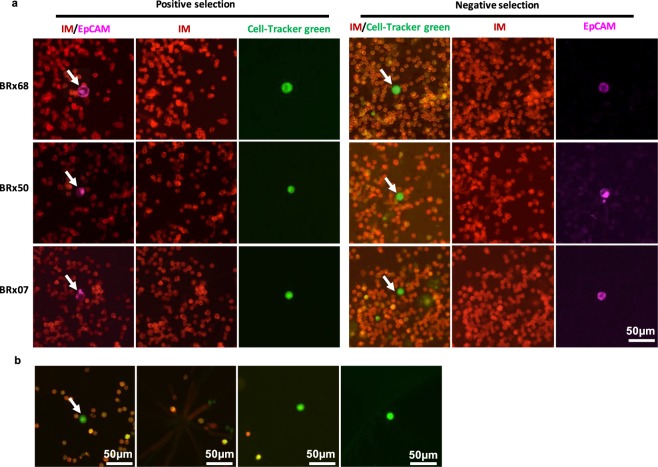
Evaluation of the PIC&RUN assay for CTC detection. (**a**) Specificity of the PIC&RUN approach for the detection of CTCs. Left panel shows positive selection module on CTC detection using Cell-Tracker green pre-stained CTCs spiked into healthy volunteers’ blood. Identified CTCs are positive for EpCAM staining (Magenta, arrows) and negative for IM staining (red). The third column confirms that the detected CTCs are Cell-Tracker green^+^ (green). Right panel shows negative selection module on CTC detection using EpCAM pre-stained CTCs spiked into healthy volunteers’ blood. Identified CTCs are positive for Cell-Tracker green and negative for IM (arrows). The third column confirms that these detected CTCs are EpCAM^+^. (**b**) Isolation of single live CTCs. From left to right: a CTC (arrow) that is positive for Cell-Tracker green (green) and negative for IM (red); the same field of view after picking the CTC; the same CTC after deposition in a glass bottom PCR tube; a CTC after two picks and transferred into 96 well plate.

For positive selection, CTCs pre-stained with Cell-Tracker green (ranging from 313 to 695 cells) were spiked into 7.5 ml of blood from healthy volunteers and detected as EpCAM^+^/IM^−^ cells via positive selection protocol with an average of 78% detection rate. We confirmed the identity of the detected CTCs via Cell-Tracker green staining and found that 98% of the detected CTCs are Cell-Tracker green^+^, 2% (9 out of 438) were Cell-Tracker green^−^ (false positive), and 3% (19 out of 560) of the spiked-in cells were EpCAM^−^/IM^−^/Cell-Tracker green^+^ (false negative) (Supplementary Table 1). The discrepancy between these data and our initial testing of AccuCyte detection efficiency of live CTCs may be due to cell loss during the staining procedure or error related to large number of spiked cells. To simulate the rare number of CTCs in patients’ blood, Cell-Tracker green pre-stained CTCs were precisely counted on 8 chamber slides and single BRx68, BRx50 or BRx07 cells or 14 pre-stained CTCs (BRx68) were picked using RareCyte needle and spiked into 7.5 ml of blood from healthy volunteers and processed by positive selection method. We were able to detect the single spiked CTCs in three independent experiments and 12/14 CTCs in one experiment, without any false positive or false negative CTCs (Table 2). Identities of the detected CTCs were confirmed by the positive Cell-Tracker green staining (Fig. 2a).

**Table 2:** Sensitivity of positive and negative selection approaches for detection of live CTCs.

	Positive Selection				Negative Selection			
No. Spiked cells	1	1	1	14	1	1	1	24
No. Detected cells	1	1	1	12	1	1	1	24

To evaluate the capture rate of the negative selection approach, CTCs were pre-stained with anti-EpCAM antibody. Single or 24 EpCAM^+^ CTCs were precisely picked as mentioned above and spiked into 7.5 ml of healthy volunteers’ blood, which was then processed with the negative selection approach. 24 out of 24 CTCs, and single CTCs in three independent experiments were detected (Table 2). All detected CTCs were confirmed through their pre-stained EpCAM signal (Fig. 2a).

To evaluate specificity of the negative selection module, 8 blood samples from 3 different healthy volunteers were processed. False positive events were detected in 7 out of 8 blood samples from the three different healthy volunteers ranging from 2 to 6 (Supplementary Table 2). These events may be immune cells with a very weak IM staining or other normal circulating cells. The presence of non-hematologic cells circulating in healthy individuals’ blood has been previously reported but not explained^8,17,44,45^. Thus, CTCs detected by negative selection protocol should be further validated as the case for all other negative depletion based CTC platforms^21,46–51^ or used exclusively for downstream applications that will provide further validation, such as CTC culture or molecular analyses.

### Optimization of single live CTC retrieval

Using an interactive manipulation of RareCyte needle, we optimized a protocol for picking single live CTCs from buffy coats, which showed over 80% successful picks with around 100 to 150 WBCs cross contamination in one pick (Fig. 2b). For single cell molecular analyses, it is crucial that CTCs are isolated individually without contaminating immune cells. Ultra-pure single CTCs were successfully retrieved by adding a second pick step (Fig. 2b and Supplementary Fig. 4). This ultra-pure picking protocol provides a capability to retrieve pure single viable CTCs in 0.5 µL final volume of any buffer or media compatible with downstream studies.

### Positive CTC selection is compatible with single cell transcriptomic analyses

**Figure 3:**
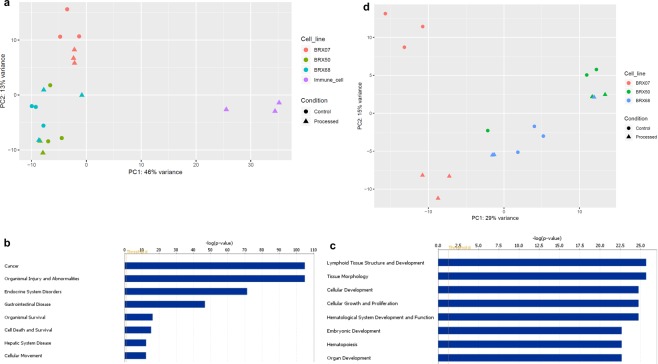
Single cell RNA sequencing analyses. (**a**) PCA plot for single CTCs and immune cells identified using positive selection protocol. The CTC population includes cells separated using serial dilution (Control) and cells which were spiked in healthy volunteers’ blood and isolated individually using positive selection protocol (processed). (**b**,**c**) IPA prediction of functions based on DEGs of upregulated genes in CTCs (**b**) or immune cells (**c**). **(d)** PCA plot for processed Vs. Control CTCs.

We then tested applicability of the positive selection approach for single cell transcriptomic analysis. Cells from three CTC lines (BRx07, BRx50 and BRx68) were spiked into healthy volunteers’ blood and processed for CTC identification using positive selection protocol. Three EpCAM^+^/IM^−^ CTCs were picked from each cell line using our two-step picking protocol yielding ultra-pure single CTCs. Three IM^+^/EpCAM^−^ immune cells were also picked individually. Three CTCs from each CTC line were isolated using serial dilution and served as control. Principal Component Analysis (PCA) plots of RNA sequencing data of these single cells showed a clear separation between immune cells and all CTCs, regardless of the isolation procedure (RareCyte vs. serial dilution) (Fig. 3a). Ingenuity Pathway Analysis (IPA) of differentially expressed genes (DEGs) between the isolated CTCs and immune cells showed that the top predicted function of up-regulated genes in CTCs is “cancer” compared to “lymphoid tissue structure and development” for the isolated immune cells (Fig. 3b,c). This result further validated the precision of our positive selection protocol and demonstrated that the identified and picked cells are compatible with single cell transcriptomic analysis.

To test if processing through the positive selection protocol influences gene expression profiles, we ran PCA plot for processed versus control CTCs (Fig. 3d). This showed that, our positive detection and retrieval of live CTCs has no obvious effect on the transcriptomes of BRx50 or BRx68 CTC lines. However, processed cells of BRx07 line clustered together away from control cells, suggesting an effect of processing on this cell line. This may attribute to the effect of antibody in this particular line or the possibility that cells may have experienced attack from immune cells from a different individual. This latter issue would not exist in real patient samples.

### Negative CTC selection is compatible with CTC *ex vivo* culture

**Figure 4:**
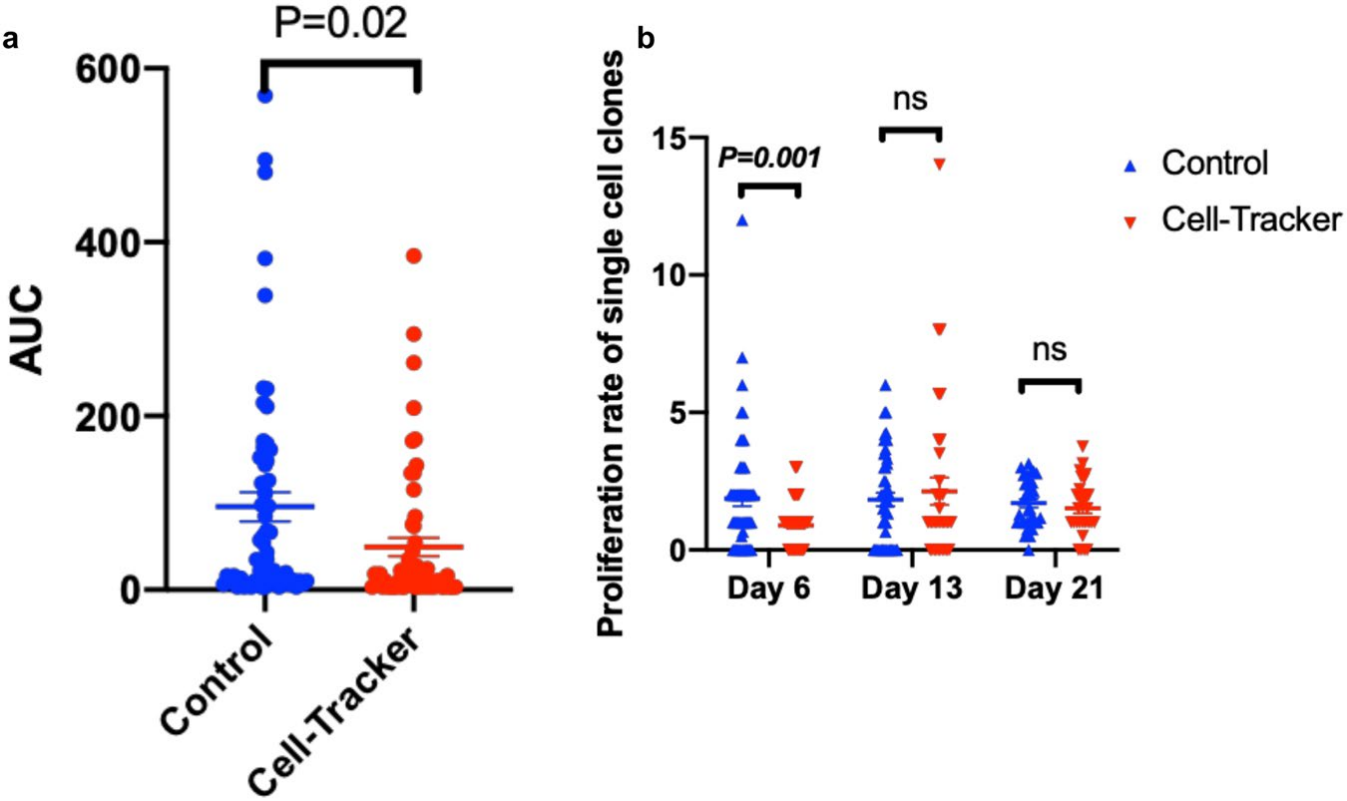
*Ex vivo* culture of single CTCs isolated using negative selection. (**a**) Area under the curve (AUC) measured for single cell clones over 21 days in CTCs isolated via serial dilution (control) or via negative selection protocol of spiked-in CTCs (Cell-Tracker). (**b**) Proliferation rates of single cell clones over time from three independent experiments; Mean ± SEM.

The applicability of negative selection approach for *ex vivo* culture was tested in three independent experiments. CTCs were spiked into healthy donors’ blood and processed using the negative selection protocol with Cell-Tracker green as a LCD. In each experiment, a range of 16 to 24 Cell-Tracker green^+^/IM^−^ cells were picked and cultured individually for 3 weeks. Similar numbers of cells were isolated individually using serial dilution and were cultured in parallel to serve as controls. The mean of area under curve (AUC) of single cell clones’ proliferation over time in the control group was significantly higher than that in the Cell-Tracker green group (Fig. 4a). This difference is caused by a significant delay in cell proliferation of the cell-Tracker green^+^ cells during the first week of culture. However, their proliferation rates were comparable to control cells at weeks 2 and 3 (Fig. 4b). The delay observed in the Cell-Tracker green group may be a consequence of the handling process or an immune reaction of the healthy volunteers’ blood against spiked cells.

### Detection and isolation of CTCs from breast cancer patients

**Figure 5:**
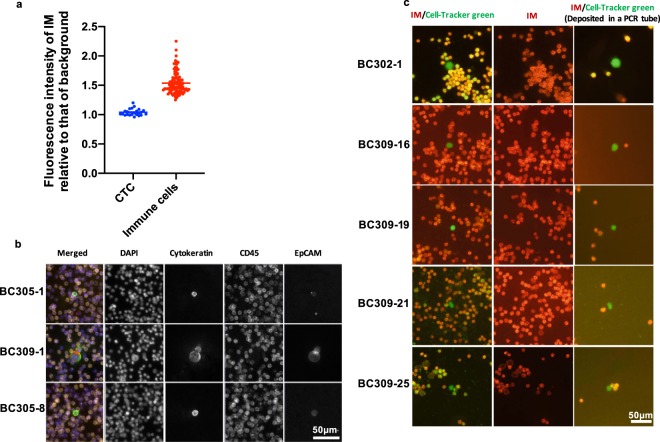
Detection of CTCs in the blood of breast cancer patients. (**a**) Quantification of the relative fluorescence signal of IM staining in immune cells and spiked-in CTCs. (**b**) Representative images for CTCs detected in patients using AccuCyte-CyteFinder system. (**c**) Representative images of CTCs detected and picked using PIC&RUN (negative selection).

We evaluated the performance of the PIC&RUN assay (live CTCs) against the AccuCyte-CyteFinder system (fixed CTCs)^42^ in advanced breast cancer patients’ samples. The enumeration of CTCs was assessed in 14 patients using both AccuCyte-CyteFinder and PIC&RUN (negative selection). In 5 of the 14 patients, CTCs were also quantified using PIC&RUN (positive selection). In order to unbiasedly determine the negativity of IM, we quantified the fluorescence of IM staining relative to the background in immune cells and in spiked-in CTCs and determined less than 1.2 as a threshold for the negativity in IM channel (Fig. 5a).

PIC&RUN (Negative selection) showed much higher number of CTC-positive patients and higher CTC counts per patient compared to both AccuCyte-CyteFinder and PIC&RUN (positive selection) (Table 3). Interestingly, in the 5 patients who were tested using the three methods, they were negative using both AccuCyte-CyteFinder and PIC&RUN (positive selection), whereas the PIC&RUN (negative selection) showed 3 out of 5 patients were positive with a range of 4 to 13 CTCs (Table 3). CTCs detected by the AccuCyte-CyteFinder system as well as the PIC&RUN assay varied in size and morphology (Fig. 5b,c). The high CTC numbers detected by negative selection agree with the level of false positive events detected in the healthy volunteers’ blood using negative selection as discussed above. This may also indicate the possibility of the presence of CTC’s subpopulations beyond the detection window of both the AccuCyte-CyteFinder system (which only detects epithelial CTCs) and the PIC&RUN (positive selection) which detects only CTCs positive for EpCAM, HER2 and/or EGFR. Studies showed that CTCs are heterogeneous and epithelial and mesenchymal CTCs could be present in the blood of the same patient^23,24,26,52^. Although the number of patients in our study is too small to draw a conclusion, there was a trend of association between CTC counts, measured by PIC&RUN (negative selection), and patients’ prognosis. The highest number of CTCs was found in the only patient (BC309) who had a progressing disease under treatment after receiving 5 lines of therapy. The second highest number of CTCs was in a patient (BC305) with the highest number of metastatic organs, despite having stable disease at the time of blood draw (Table 3).

**Table 3:** CTC counts using AccuCyte-CyteFinder and PIC&RUN in breast cancer patients.

Patient ID	Age at diagnosis	ER/PR/HER2 Status	Disease Status at time of blood draw	Disease Status at last visit	Days from blood draw to last visit	Lines of Treatment	Metastatic sites	CTC counts in 7.5 ml of blood		
								AccuCyte-Cytefinder	PIC&RUN	
									Negative selection	Positive selection
BC299	45	ER^−^/PR^−^/HER2^−^	NA	CR^t^	154	1	NA	0	3	NA
BC303	61	ER^+^/PR^+^/HER2^−^	SD	SD^tt^	121	1	Bone	0	3	NA
BC304	50	ER^−^/PR^−^/HER2^+^	SD	PD^ttt^	119	3	Sternum, LN*, chest wall	0	3	NA
BC305	53	ER^−^/PR^−^/HER2^+^	SD	NA	70	1	Bone, LN, chest wall, lung, skin,muscle invasion	14	17	NA
BC306	31	ER^+^/PR^+^/HER2^−^	SD	SD	107	1	Bone	0	6	NA
BC307	63	ER^+^/PR^+^/HER2^−^	SD	SD	93	1	Bone, pleura, LN	2	5	NA
BC308	58	ER^+^/PR^+^/HER2^−^	SD	SD	63	1	Bone	0	4	NA
BC309	54	ER^−^/PR^−^/HER2^+^	PD	PD	56	5	Breast, LN, skin, brain	4	24	NA
BC310	47	ER^−^/PR^−^/HER2^−^	NA	NA	23	0	LN, Skin, chest wall, pleural effusion	0	4	NA
BC311	44	ER^+^/PR^+^/HER2^−^	SD	SD	0	1	Visceral- liver, lungs	0	13	0
BC312	66	ER^+^/PR^+^/HER2**	SD	SD	0	1	Bone	0	0	0
BC314	48	ER^+^/PR^+^/HER2^−^	SD	CR	30	1	LN	0	4	0
BC315	52	ER^−^/PR^−^/HER2^+^	PR^tttt^	SD	21	1	Skin, LN	0	0	0
BC316	39	ER^+^/PR^+^/HER2^−^	SD	SD	1	1	Gastric, ovaries, fallopian tube, cervix	0	12	0

*LN = lymph node.

** = equivocal.

^t^CR = complete response, ^t t^ SD = stable disease, ^t t t^  PD = progressive disease, ^tttt^ PR = partial response.

In conclusion, our PIC&RUN approach is a novel assay for the detection and isolation of live single CTCs with two modules (positive and negative selections), allowing downstream single cell transcriptomic analyses and *ex vivo* culture of single CTCs. The limitation of our protocol is the relatively long processing time (due to semi-automatic scanning) which allows us to process up to two patient samples per day compared to 20 samples using the CD45 auto- magnetic-activated cell sorting (MACS) depletion protocol^46^. Although the semi-automatic scanning for CTCs in this assay allows for a precise adjustment of the focus for every scanned field, getting this approach fully automated may save time and efforts. Due to a long processing time and relatively more false positive events in the negative selection mode than positive-selection based methods, it is not suitable at current stage for CTC enumeration solely, but rather for research purposes, such as single cell molecular analysis or *ex vivo* culture, which can further confirm the CTC identity afterwards. Future incorporation of validated surface markers for other potential non-hematopoietic, non-tumor cells that have been reported in cancer patients, such as endothelial cells^53^ or mesenchymal cells^54^, may further improve the specificity. Despite these limitations, the PIC&RUN (negative selection) assay has a high capture efficiency comparable to or higher than other CD45 depletion methods such as CTC-iChip (97%)^21^, the microfluidic NegCTC-μChip (83.1%)^47^, deterministic lateral displacement microfluidic structure with MACS (85.1%)^48^, Myltenyi-anti-CD45 microbeads (24%)^49^, Dynabeads-anti-CD45 (97%)^49^, and CanPatrol^TM^ (80%)^50^, spiral microfluidics (80%)^51^ and autoMACS (70–88%)^46^. Moreover, PIC&RUN showed a high sensitivity, being able to detect one cell in 7.5 ml of blood. Using a 2-steps picking procedure, PIC&RUN can reach purity at the single cell resolution—the only method thus far detecting and isolating single viable CTCs in one platform.

## Methods

### Patient samples

A total of 14 breast cancer patients (Stages III and IV) and 3 healthy volunteers were enrolled. Blood samples were collected from all subjects at either Los Angeles-County + University of Southern California (USC) Medical Center or the USC Norris Comprehensive Cancer Center. All experiments were performed in accordance with protocol No. 1B-11-1 approved by the institutional review board at USC. Informed consents were obtained from all participants and/or their legal guardian/s. Approximately 2 × 10 ml of blood was collected (after discarding the first few mls of blood to avoid cross contamination from skin or blood vessels’ cells from venipuncture.

### Cell culture and spike-in experiments

We have previously established 6 breast cancer patient derived CTC lines^22^. In this study we used the CTC lines BRx68, BRx50 and BRx07 for all spike-in experiments and immunofluorescence (IF) staining optimization. CTC lines were maintained in RPMI 1640 media (Gibco) supplemented with EGF (20 ng/ml) (Peprotech), basic FGF (20 ng/ml) (Peprotech), B27 (10 ml), and Antibiotic-antimycotic (Life Technologies), in 24 well ultralow attachment tissue culture plates (Corning) at 37 °C, 5% CO_2_ and 4% O_2._ For spiking low numbers of cells, CTCs were fluorescently labeled live with either DiO (ThermoFisher Scientific), Cell-Tracker green (ThermoFisher Scientific), ViaFluor (Biotium) or antibody against EpCAM, and drawn into 8 chamber glass slides (VWR). Single, 14 or 24 cells were picked using RareCyte needle, deposited into an 8 chamber slide containing fresh media, and collected and spiked into the blood. Slides were rescanned for left over cells to confirm that the precise number of cells was spiked into the blood. For capture efficiency experiments using large numbers of cells, GFP or DiO positive CTCs were counted using hemocytometer slide and the volume of media containing the required numbers of cells was calculated.

### CTC enrichment using AccuCyte

Blood samples were processed using AccuCyte as previously described^41^. Briefly, 7.5 ml of blood was added to each AccuCyte Separation Tube (RareCyte) and tubes were centrifuged at 3000 g for 25 minutes (min). After centrifugation, a brass ring clamp (CyteSeal) was applied using a CyteSealer (RareCyte) to each tube. Plasma was then aspirated and 4 ml of displacement solution (RareCyte) was added to each tube. An EpiCollector (RareCyte) with an isolation tube pre-filled with 160 µL of isolation buffer (RareCyte) was placed on the top of each separation tube. The whole system was centrifuged for 20 min at 1000 g (ThermoFisher Scientific) and buffy coats were then collected into either 800 µl transfer fluid containing a non-formalin fixative (RareCyte) or 1 ml of CTC media and transferred to 1.8 ml eppendorf tubes for further investigation. Buffy coats resuspended in transfer fluid are kept at RT for 10 min then were spread on glass slides (8 slides per blood sample). Prepared slides were air dried for 30 minutes and stored in −20 °C until the day of staining.

### Detection of CTCs using AccuCyte-CyteFinder system

Slides were fluorescently stained with the 4D staining kit (RareCyte) which contains antibodies against cytokeratin, EpCAM, and CD45 plus DAPI using the automated staining instrument (BOND RXm, Leica Biosystem). Stained slides were automatically scanned using the fluorescence platform of the RareCyte and images were captured at 10x objective magnification. Slide images were analyzed with integrated image analysis software that automatically analyzes the images to find cytokeratin and/or EpCAM positive cells which are CD45 negative.

### Positive selection staining

Buffy coats obtained from AccuCyte diluted in 1 ml of media were stained with a cocktail of PE-CF594 conjugated antibodies against IM and a cocktail of AlexaFluor647 conjugated antibodies against CSMs for 30 min at 37 °C. The IM antibody cocktail included anti-CD45, anti-CD14 and anti-CD16 antibodies (BD Biosciences) at a dilution of 1 in 20. CSM antibody cocktail in spike in experiments is anti-EpCAM (Cell Signaling) at a dilution of 1 in 100, whereas in patients’ samples anti-HER2 (Biolegend) and anti-EGFR (Biolegend) antibodies were added to the cocktail. Cells were then transferred to 50 ml Falcon tubes and washed in 50 ml of CTC media. Tubes were centrifuged for 20 min at 200 g. After centrifugation, 25 ml of the supernatant was discarded carefully from each tube without disturbing cell pellets. An additional 25 ml of fresh CTC media was added, and tubes were centrifuged for an additional 5 min. All supernatant was removed except 24 ml of media at the bottom of each tube. Cells were pipetted up and down using 25 ml serological pipettes to obtain single cell suspensions. Cells were then plated on 4 two-well cyteslides (Rarecyte) at 3 ml/well and incubated for 30 min at 37 °C. After incubation, slides were scanned for IM^−^/CSM^+^ cells (CTCs).

### Negative selection staining

Buffy coats were stained with a cocktail of IM antibodies as described in the positive selection staining procedure and a live cell dye (LCD). Three LCDs were tested: DiO, Cell-Tracker green and ViaFluor. For both Cell-tracker green and DiO, cells were stained simultaneously with the IM antibodies cocktail for 30 min at 37 °C. For ViaFluor, cells were stained first with Viafluor for 15 min then 1 ml of fresh media was added, and cells were incubated for 5 min at 37 °C. After incubation, cells were centrifuged for 20 min at 200 g and the supernatant was removed. Cells were re-suspended in 1 ml of fresh media containing a cocktail of IM antibodies and incubated for 30 min at 37 °C. After incubation, cells were washed, plated on cyteslides, and scanned using RareCyte.

### Detection of CTCs

For both detection approaches, stained buffy coats were seeded on polyhema coated cyteslides and scanned semi-automatically using RareCyte fluorescence. Three ml of stained buffy coat was added to each chamber of the cyteslide. Slides were then incubated for 30 min at 37 °C for cells to settle. Before cell retrieval, the ceramic tip (40 µm) Rarecyte needle was primed and calibrated as previously described^41^. For positive selection, slides were scanned using the CSM fluorescent channel followed by IM channel to exclude the IM signal for the identified targets. For negative selection, slides were scanned using two fluorescent channels simultaneously.

### Retrieval of live CTCs

When a CTC was detected, the needle was positioned right on top of the cell of interest with a 0.2 mm distance between the tip of the needle and the bottom of the slide. The cell was then drawn with a draw volume of 50 nL to 0.5 µL, and the needle was moved to deposition position, where a PCR tube with 50 µL of media had been placed. The needle was then moved down to a distance of 0.6 mm from the bottom of the tube and the cell was expelled down. PCR tubes were pulse spun and their contents were moved to a well of a 96 well GravityTRAP ultra low plate (InSphero).

### Single-cell RNA-sequencing workflow

CTCs were spiked in healthy volunteers’ blood and processed using AccuCyte. Buffy coats were prepared using positive selection protocol and scanned for CTCs using RareCyte fluorescence. Single CTCs and single immune cells were retrieved using RareCyte needle. Isolated single cells were processed using the SMARTer chemistry (SMART Seq v4 Ultra Low Input RNA Kit for Sequencing, Takara Clontech) according to manufacturer’s instructions to generate single-cell cDNA libraries for mRNA sequencing. All cDNA samples were run on a TapeStation system (High Sensitivity D5000 DNA Analysis Kit as per manufacturer’s protocol). cDNA libraries were prepared using the Nextera XT DNA Library Prep Kit (Illumina) with Nextera index kit index 1 (i7) and index 2 (i5) adapters. Libraries were sequenced on an Illumina NextSeq. 500 to obtain 75 bp-long single-end reads.

RNA-sequencing reads were trimmed for Nextera and Illumina adapter sequences using Trim Galore under default parameters. Trimmed reads were then mapped to the human genome build GRCh37 from Ensembl (ftp://ftp.ensembl.org/pub/grch37/current/fasta/homo_sapiens/dna/Homo_sapiens.GRCh37.dna_sm.primary_assembly.fa.gz) using STAR under optimized parameters for single-end sequenced data. Aligned reads were then counted via featureCounts^55^ and piped into DESeq. 2^56^ for normalization to sequencing depth and downstream analysis. For purposes of producing the PCA plot, count data was transformed via the vst function to eliminate the experiment-wide trend of variance over mean and the plot was produced using ggplot2. The contrast function was used to compare all samples under the Cell Type category “CTC” to samples under the “Immune cell” category and vice versa. Genes with a False Discovery Rate (FDR) of 0.05 and log2 fold change of >2 were piped into IPA to predict function of each cell group.

### *Ex vivo* culture of single CTCs

Single CTCs were cultured in GravityTRAP ultralow 96 well plates in CTC media at 37 °C, 5% CO2 and 4% O2 for 3 weeks. Media was replaced every 3–4 days and cells were counted every week. Proliferation rates of single cell clones were calculated as number of cells at a specific time point divided by that at the previous time point for each clone.

### Statistical analyses

Statistical analysis was performed using GraphPad Prism (version 8.2.0). Data are represented as mean ± SEM. Data distribution was tested using the Shapiro-wilk test. If data were normally distributed, statistical significance was measured using parametric testing, with standard independent samples t-tests. If data were not normally distributed, non-parametric Mann-whitney U test was used to assess the difference between test and control samples. Differences were considered statistically significant if the probability value (p) was ≤0.05.

## Supplementary information

Suplementary data all

## Data Availability

RNA sequencing data is available upon request following publication.
