# Author Correction: PIC&RUN: An integrated assay for the detection and retrieval of single viable circulating tumor cells

**DOI:** 10.1038/s41598-020-60008-3

**Published:** 2020-02-13

**Authors:** Mohamed Kamal, Shahin Saremi, Remi Klotz, Oihana Iriondo, Yonatan Amzaleg, Yvonne Chairez, Varsha Tulpule, Julie E. Lang, Irene Kang, Min Yu

**Affiliations:** 1 Department of Stem Cell Biology and Regenerative Medicine, Keck School of Medicine of the University of Southern California, Los Angeles, CA 90033 USA; 2 USC Norris Comprehensive Cancer Center, Keck School of Medicine of the University of Southern California, Los Angeles, California 90033 USA; 3 Department of Zoology, Faculty of Science, University of Benha, Benha, Egypt; 4 MS Biotechnology program, California State University Channel Islands, Camarillo, CA 93012 USA; 5 Department of Medicine, Keck School of Medicine of the University of Southern California, Los Angeles, California 90033 USA; 6 Department of Surgery, Keck School of Medicine of the University of Southern California, Los Angeles, California 90033 USA

Correction to: *Scientific Reports* 10.1038/s41598-019-53899-4, published online 25 November 2019

This Article contains a typographical error in the Introduction where,

“Currently, to isolate single live CTCs, additional purification steps, such as the DEPArray^34,35^, Fluidigm C1^36–39^, ALS cell-Selector^40^ or single-cell micro-manipulation, are typically used.”

should read:

“Currently, to isolate single live CTCs, additional purification steps, such as the DEPArray^34,35^, Fluidigm C1^36–39^, ALS CellCelector^40^ or single-cell micro-manipulation, are typically used.”

