# Supplementary material for: PIC&RUN: An integrated assay for the detection and retrieval of single viable circulating tumor cells
Source: Sci Rep. 2019 Nov 25;9:17470. doi: 10.1038/s41598-019-53899-4 (PMC6877641; doi:10.1038/s41598-019-53899-4)
Supplement: Supplementary file 1 — Suplementary data all [file 41598_2019_53899_MOESM1_ESM.pdf]

Supplementary Figs and Tables

PIC&RUN: An integrated assay for the detection and retrieval of single viable circulating tumor cells

Mohamed Kamal[1,2,3], Shahin Saremi[1,2,4], Remi Klotz[1,2], Oihana Iriondo[1,2], Yonatan Amzaleg[1,2], Yvonne Chairez[1,2], Varsha Tulpule[2,5], Julie E. Lang[2,6], Irene Kang[2,5], and Min Yu[1,2*]

[1]Department of Stem Cell Biology and Regenerative Medicine, Keck School of Medicine of the University of Southern California, Los Angeles, CA, 90033, USA. [2]USC Norris Comprehensive Cancer Center, Keck School of Medicine of the University of Southern California, Los Angeles, California, 90033, USA. [3]Department of Zoology, Faculty of Science, University of Benha, Benha, Egypt. [4]MS Biotechnology program, California State University Channel Islands, Camarillo, CA 93012. [5]Department of Medicine, Keck School of Medicine of the University of Southern California, Los Angeles, California, 90033, USA. [6]Department of Surgery, Keck School of Medicine of the University of Southern California, Los Angeles, California, 90033, USA.

[*]Correspondence: minyu@med.usc.edu

A. Supplementary Figures

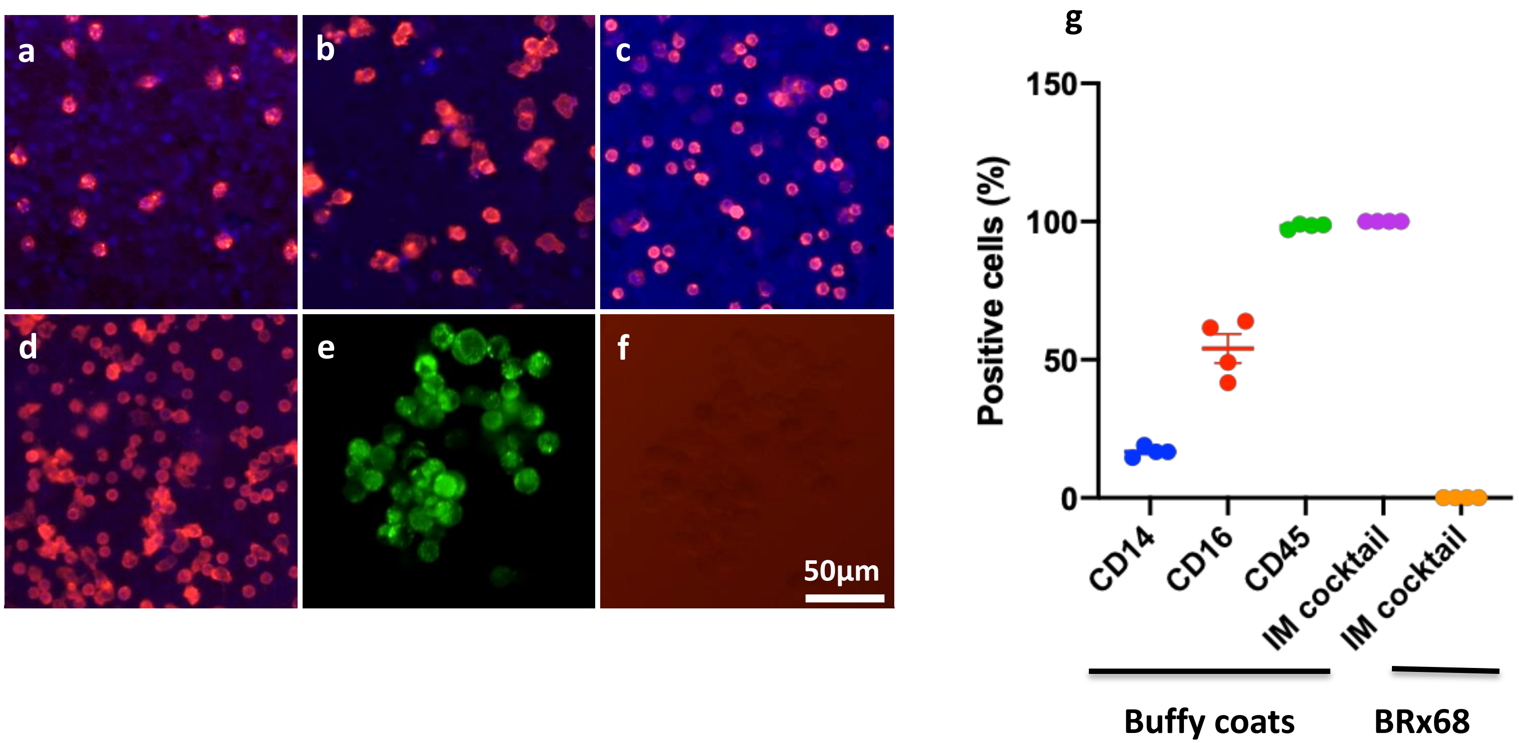

Supplementary Figure 1. Sensitivity and specificity of immune cell markers. Buffy coats from healthy volunteers' blood were stained live with the nuclear stain Hoechst33342 (blue) and immune markers (red) with antibody against CD14 (a), CD16 (b), CD45 (c), or a cocktail of the three antibodies (d). BRx68 CTCs were double stained with DiO (green) (e) and IM antibodies cocktail (red) (f). Percentage of positive cells for each marker individually (CD14: 16.73 ± 0.9, CD16: 54.03 ± 5.4, CD45: 98.36 ± 0.4) or combined (100 ± 0.0) were calculated from a total of 500 to 1200 cells from 4 different images (Mean±SEM) (g).

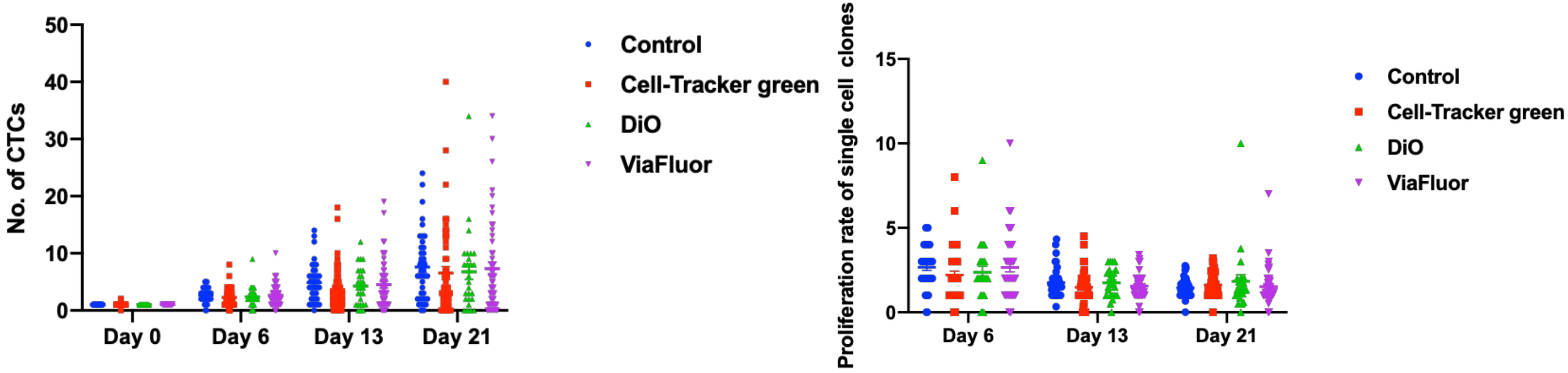

Supplementary Figure 2. Effects of live cell dyes on proliferation of single cells. Graphs showing numbers of cells (left) or proliferation rates (right) in single CTC clones over time. Single CTCs were isolated via serial dilution of unstained CTCs (control) or CTCs stained with either Cell-Tracker green, DiO, or ViaFluor. Cells were cultured *ex vivo* for three weeks and number of cells in each single cell clone was counted each week in three independent experiments; Mean±SEM.

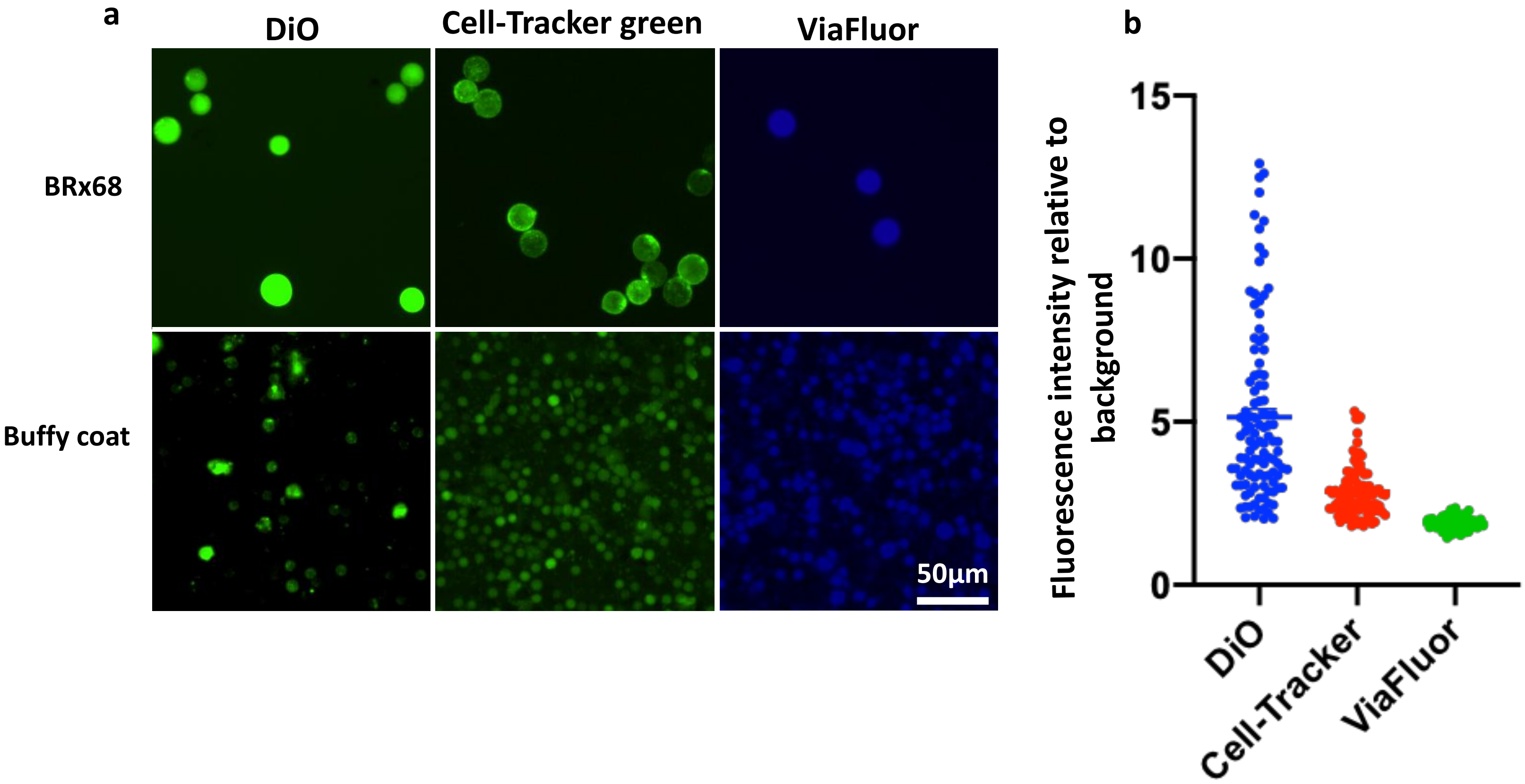

Supplementary Figure 3. Live cell dye staining. a) Representative images of BRx68 CTC line (top panel) and buffy coats from healthy volunteers' blood (bottom panel) stained with DiO (left), Cell-Tracker green (middle), and ViaFluor (right). b) Graph showing relative fluorescence intensity to background measured using ImageJ in 100 cells for each live dye.

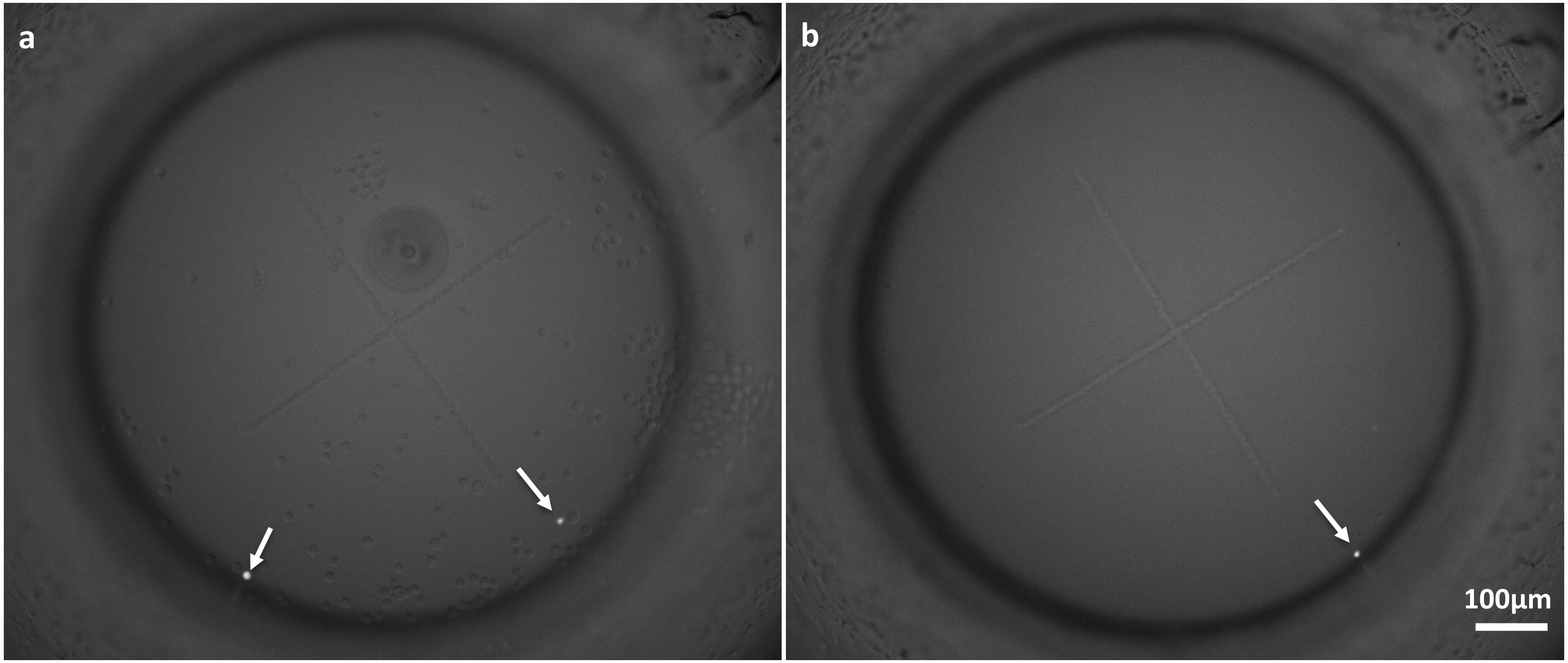

Supplementary Figure 4. Retrieval of live single cells. GFP$^+$ CTC mixed with none GFP CTCs were plated on cyteslides and GFP$^+$ CTCs were detected and picked using RareCyte needle. a) Bottom of a PCR deposition tube after first pick showing two GFP$^+$ cells (arrows) with over 100 none GFP cells. b) Bottom of a PCR deposition tube after second pick showing ultra-pure single GFP$^+$ cell (arrow).

B. Supplementary Tables

Supplementary Table 1: CTC capture efficiency by positive selection

Exp No.	No. Spiked cells	No. Detected cells (%)	No. False positive cells (%)	No. False Negative cells (%)
1	313	237 (76)	8 (3.4)	25 (8)
2	695	551 (79)	11 (2)	21 (3)
3	673	525 (78)	8 (1.5)	10 (1.5)
Average	560	438 (78)	9 (2)	19 (3)

Supplementary Table 2: False positive CTC counts using PIC&RUN (negative selection) in healthy individuals' blood.

Individual	Sample	No. false positive cells
A	1	0
A	2	0
A	3	3
A	4	3
A	5	3
B	1	2
B	2	6
B	3	2
C	1	2
**Average**		**2.33**
**range**		**0-6**
